# Recognition of bacteria named entity using conditional random fields in Spark

**DOI:** 10.1186/s12918-018-0625-3

**Published:** 2018-11-22

**Authors:** Xiaoyan Wang, Yichuan Li, Tingting He, Xingpeng Jiang, Xiaohua Hu

**Affiliations:** 1 School of Computer, Central China Normal University, Wuhan, Hubei China; 2 College of Computing and Informatics, Drexel University, Philadelphia, PA USA

**Keywords:** Spark, Named entity recognition, Text mining, Microbial interactions

## Abstract

**Background:**

Microbe plays a crucial role in the functional mechanism of an ecosystem. Identification of the interactions among microbes is an important step towards understand the structure and function of microbial communities, as well as of the impact of microbes on human health and disease. Despite the importance of it, there is not a gold-standard dataset of microbial interactions currently. Traditional approaches such as growth and co-culture analysis need to be performed in the laboratory, which are time-consuming and costly. By providing predicted candidate interactions to experimental verification, computational methods are able to alleviate this problem. Mining microbial interactions from mass medical texts is one type of computational methods. Identification of the named entity of bacteria and related entities from the text is the basis for microbial relation extraction. In the previous work, a system of bacteria named entities recognition based on the dictionary and conditional random field was proposed. However, it is inefficient when dealing with large-scale text.

**Results:**

We implemented bacteria named entity recognition on Spark platform and designed experiments for comparison to verify the correctness and validity of the proposed system. The experimental results show that it can achieve higher F-Measure on the comparison of correctness. Moreover, the predicting speed is much faster than the previous version in large-scale biomedical datasets, and the computational efficiency is improved remarkably by about 3.1 to 6.7 times.

**Conclusions:**

The system for bacteria named entity recognition solves the inefficiency of the previous proposed system on large-scale datasets. The proposed system has good performance in accuracy and scalability.

## Background

Microbes are almost everywhere in the global environment. Soils, plant, water and animals are the environment of one or more microbial communities. A variety of microbial communities formed by the aggregation of different proportions microorganisms are commonly referred to as the microbiome. Microbes in the microbiome frequently interact with other members of the community, and these interactions reflect the overall structure and function of the microbial community [1]. Microbes are closely related to host health. Unbalance in microbial communities will lead to a variety of diseases. For example, the microbiome affects the host by making it susceptible to central nervous system autoimmune diseases [2]. Studying the relationships between microbes and diseases provides a new potential to cure a number of diseases. For instance, gastrointestinal microflora can affect fat storage, and thus recovering gut microflora to a healthy state which is helpful for solving the obesity-related problems [3]. In the past 10 years or so, researchers have developed a variety of computational methods for mining a large number of microbial interactions from metagenome abundance data. For example, using the Fisher’s exact test to infer whether species co-occur or co-exclusion from spatial metagenomic survey data [4], using the Spearman, Pearson and other correlation coefficients to identify the correlation between microbial species, or using the LSA algorithm to infer directional interactions from temporal metagenomic data [5]. On the other hand, a large number of microbial interactions validated by many biological experiments are reported in mass biomedical literature and which are often overlooked. Mining these interactions and collating them into a database will create a valuable resource for current research. As one of the main ways to show results and exchange academic results, biomedical literatures accumulate rapidly and its scale is far exceeding those of other disciplines. In particular, there are over 2 million articles related to bacteria studies. How to effectively use these massive data to quickly and accurately discover valuable information are becoming an important part of current research. There are still few studies on how to find out the interactions between microbes from mass biomedical literature. Freilich et al. [6] studied the interactions between microbes based on the co-occurrence of species in the text and constructed an approximate model of the bacterial ecosystem. Lim et al. [7] used support vector machine(SVM) to classify and determine whether there is positive or negative interaction between the given microbial species, which greatly reduces the manual annotation workload, but cannot determine the mode or direction of interactions.

One of the basic tasks of text mining is named entity recognition, which aims to automatically identify the proper nouns. The identification of microbial named entities remains a challenging task, due to the lack of standard corpus, the emergence of new named entities, the existence of phenomena that one entity with different writings and long entities nesting short entities. Named entity recognition (NER) approaches mainly include rule-based methods, dictionary-based methods, and machine learning-based methods. The current mainstream method for NER is machine learning, and of them conditional random field (CRF) is an excellent algorithm among them. In our previous work [8], we manually annotated datasets and proposed a bacteria named entity recognition system with good performance based on the dictionary and CRF. However, for the massive biomedical literature that needs to be identified, the system will encounter a series of challenges in big data processing, including huge computational time and space requirements.

Transferring large-scale computing tasks to the distributed cluster platform has become an effective way to solve the above problems. Spark is a memory-based parallel framework, which will cache the data that will be used repeatedly to the memory to reduce the data loading time. In addition, for the given task, Spark will build a Directed Acyclic Graph (DAG) which tightly arranges calculations and calculations. Hence the framework is able to automatically optimizes tasks according to the logical relationship between operators. The same iterative machine learning algorithm runs faster in Spark than Hadoop by 10~ 100 times. [9]. Therefore, the execution efficiency of the Spark framework is relatively superior. Literature [10] proposes a parallel ant colony optimization (ACO) algorithm based on Spark for combinatorial optimization in the era of big data, which is more than 10 times faster than that based on MapReduce. Literature [11] achieves parallelized frequent item sets mining algorithm based on Spark, and compared it with the algorithm implemented based on MapReduce on a number of benchmark experiments. The experimental results show that the former has an average speed of 18 times faster than the later.

Based on the previous results [8], we proposed a parallel bacteria named entity recognition system based on Spark platform and CRF. The experiment shows that the speed of the Spark version has been greatly improved, with higher time efficiency and good scalability. This lays a foundation for the extraction of bacteria interactions from medical literature.

## Materials and methods

### Experimental environment and data sets

The experimental environment is as follows: Debian, 3.16.0–4-amd64, Intel(R) Xeon(R) CPU E5–2670 v3 @ 2.30GHz processors, 256GB RAM, Apache Spark 2.2.1, Scala-2.11.8 and JDK1.8.0_71. We built a Spark application with a Stand-alone cluster task scheduling mode on a 48-core server. The CRF algorithm used in the experiments is an open source CRF algorithm in Spark [12]. They use Adam and AdaGrad optimizer based on Spark, so it will get better performance compared with other methods [13, 14].

The datasets used are the corpus (IOB2 format) that are manually annotated in our previous work [8] for bacteria named entity recognition and the 50,000 unannotated biomedical abstracts downloaded on PubMed with the keyword “human”, “oral”, “bacteria”.

### Methods

In this paper, we mainly study the computing platform for bacteria named entity recognition based on the conditional random field and Spark. To begin with, we extracted 34 features such as word features, affix features, etc. We trained the CRF model on a training sets in Spark, and then evaluated the model’s performance on a test set. Finally, we compared the Spark version and CRF++ on single node under the same conditions to verify the efficiency of the system, and tried to apply them to large-scale unannotated corpus to compare the prediction speed of them.

### Spark computing framework

Representative batch systems include MapReduce [15], Spark [9], Pregel [16] and Trinity [17], etc. Among them, Spark is implemented in Scala language and compatible with Hadoop’s original ecosystem while overcoming the shortcomings of MapReduce in iterative computing and interactive data analysis. In addition, it has the advantages of scalability, high reliability and load balancing, and has a huge community support, so it has become the most active and efficient general computing platform for large data. Resilient Distributed Dataset (RDD) [18] is the core data structure of Spark, the scheduling order of Spark is formed by the dependency of RDD, and entire Spark program is formed by the operation of RDD. With such memory calculation mode, Spark supports machine learning and other iterative computing well and has better computational efficiency than MapReduce.

### Conditional random field

The conditional random field was first proposed by Lafferty et al. in 2001 [19], which is a discriminant undirected graph model that models the conditional probabilities according to the given observation sequence of variables. In the field of biomedicine, linear chain CRFs are generally used to process sequence labeling tasks such as named entity recognition and part-of-speech tagging and so on.

Assuming X and Y are random variables, P(Y| X) is the conditional probability distribution of Y given X. If the random variable Y constitutes a Markov random field represented by an undirected graph G = (V,E),

1 $$ \mathrm{P}\left({\mathrm{Y}}_{\mathrm{v}}|\mathrm{X},{\mathrm{Y}}_{\mathrm{w}},\mathrm{w}\ne \mathrm{v}\right)=\mathrm{P}\left({\mathrm{Y}}_{\mathrm{v}}|\mathrm{X},{\mathrm{Y}}_{\mathrm{w}},\mathrm{w}\sim \mathrm{v}\right) $$

that is, Eq. (1) holds for any node v, then the conditional probability distribution P(Y|X) is called a conditional random field.

In Eq. (1), w~v denotes all nodes w that have edges connected to node v in the graph G = (V, E), w ≠ v represents all nodes other than the node v, and Y_V_、Y_u_、Y_w_ are random variables corresponding to node v、u、w.

Assume that X = (X_1_, X_2_, …, X_n_)and Y = (Y_1_, Y_2_, …, Y_n_) are all random variable sequences represented by linear chains. If given a random variable sequence X, the conditional probability distribution P(Y| X) of the random variable sequence Y constitute a conditional random field, which means Markov Property is satisfied:

2 $$ \mathrm{P}\left({\mathrm{Y}}_{\mathrm{i}}|\mathrm{X},{\mathrm{Y}}_{1,}\dots, {\mathrm{Y}}_{\mathrm{i}-1},{\mathrm{Y}}_{\mathrm{i}+1},\dots, {\mathrm{Y}}_{\mathrm{n}}\right)=\mathrm{P}\left({\mathrm{Y}}_{\mathrm{i}}|\mathrm{X},{\mathrm{Y}}_{\mathrm{i}-1,},{\mathrm{Y}}_{\mathrm{i}+1}\right) $$

where i = 1, 2, …, n (Only one side is considered when i = 1 and n).

Then P(Y| X) is a linear chain conditional random field. In the labeling problem, X represents the input observation sequence, Y represents the corresponding output sequence or state sequence. Under the condition that random variable X is x, Y is y, the parametric form of the conditional probability is as follows:

3 $$ \mathrm{P}\left(\mathrm{y}|\mathrm{x}\right)=\frac{1}{\mathrm{Z}\left(\mathrm{x}\right)}\exp \left\{\sum \limits_{\mathrm{i},\mathrm{k}}{\uplambda}_{\mathrm{k}}{\mathrm{t}}_{\mathrm{k}}\left({\mathrm{y}}_{\mathrm{i}-1},{\mathrm{y}}_{\mathrm{i}},\mathrm{x},\mathrm{i}\right)+\sum \limits_{\mathrm{i},\mathrm{l}}{\mathrm{u}}_{\mathrm{l}}{\mathrm{s}}_{\mathrm{l}}\left({\mathrm{y}}_{\mathrm{i}},\mathrm{x},\mathrm{i}\right)\right\} $$

4 $$ \mathrm{Z}\left(\mathrm{x}\right)=\sum \limits_{\mathrm{y}}\exp \left\{\sum \limits_{\mathrm{i},\mathrm{k}}{\uplambda}_{\mathrm{k}}{\mathrm{t}}_{\mathrm{k}}\left({\mathrm{y}}_{\mathrm{i}-1},{\mathrm{y}}_{\mathrm{i}},\mathrm{x},\mathrm{i}\right)+\sum \limits_{\mathrm{i},\mathrm{l}}{\mathrm{u}}_{\mathrm{l}}{\mathrm{s}}_{\mathrm{l}}\left({\mathrm{y}}_{\mathrm{i}},\mathrm{x},\mathrm{i}\right)\right\} $$

Where t_k_ and s_l_ are eigenfunctions, their value is 1 when the feature is satisfied, 0 otherwise. λ_k_ and u_l_are the corresponding weights. Z(x)is a normalization factor, summation is done on all possible output sequences. The conditional random field is completely determined by the eigenfunction and corresponding weights. The main tasks of training are feature selection and parameter estimation. The purpose of feature selection is to choose a feature set that can express this random process, and the parameter estimation is to estimate the weights for each feature selected. The training process can be essentially attributed to the process of estimating the weight parameters of the eigenfunctions based on the principle of maximum likelihood function. When the model training is completed, the maximum likelihood distribution and model parameters are obtained. For the new observation sequence X, the most likely output sequence Y is predicted based on training model. The conditional random fields can make full use of contextual label information to achieve good labeling results.

The computational scale of the conditional random field in training is related to the size of training set, templates and the number of output tags. The sequence of input sentences in biological texts is generally very long, so there exists the problems of long time excution of optimization and large memory occupation when training on large-scale data. Research on the efficiency of CRF in handling massive data has become one of the most popular hotspots in biomedical named entity recognition. Literature [20] implements CRFs training on large-scale parallel processing systems based on multi-core and can process large data sets with hundreds of thousands of sequences and millions of features, which significantly reduces the computation time. At the same time, using a second-order Markov-dependent in the training process, the model has achieved higher accuracy; Literature [21] deals with complex computing tasks by decomposing the learning process into smaller and simpler sub-problems. It developed a core approach to learn CRF structure and parameters and speeded up the regression by using more and more parallel platforms. Literature [22] controls the number of non-zero coefficients by introducing penalties in the CRFs model. Ignoring execution time, it implements CRF’s training task on processing hundreds of output tags and up to several billion features; In literature [23], CRF-RNN, a new neural network is proposed based on mean-field approximation and Gaussian potential functions for CRFs. And they obtained the best result of the challenging Pascal VOC 2012 segmentation benchmark when applying the proposed method to the semantic image segmentation problem. Literature [24] achieves the MapReduce-based parallel training of CRFs and can ensure the correctness of the training results. Meanwhile, it greatly reduces the training time and improves the performance. Although this MapReduce-based implementation can handle large-scale training sets and feature sets, the execution efficiency is not high enough. Literature [25] converts all data into RDDs and stores them in the memory of the cluster nodes. It implements SparkCRF, a distributed CRFs running in a cluster environment. Experiments show that SparkCRF has high computing performance and good expansibility, and it has the same accuracy level as the traditional single-node CRF++.

### Design and implementation of the system

**Fig. 1:**
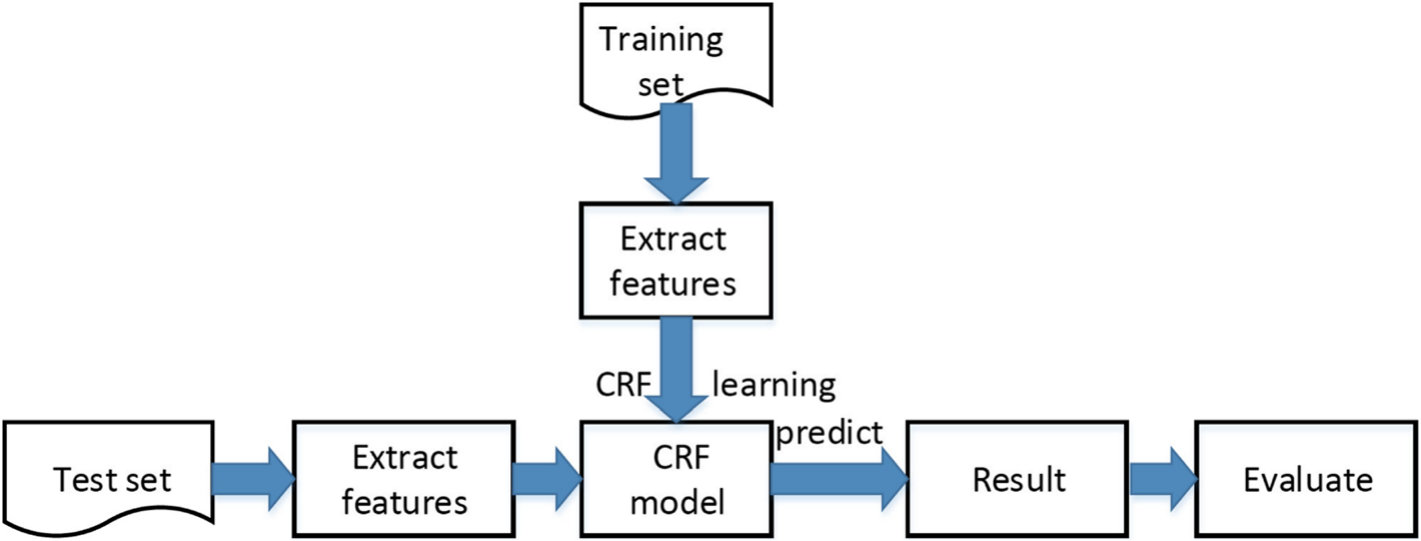
The Bacteria named entity recognition system flow chart

The proposed system is written in Scala. Firstly, we extracted the features from the data sets on the Spark platform. The features used are the optimal 34 sub-features selected by the single optimal combination method in our previous work [8], and a feature matrix was generated in the next step. The training and predicting steps were executed using the Open Source Toolkit of CRF based on Spark(We call it “Spark-CRF”). The flow chart of the bacteria named entity recognition system is shown in Fig. 1.

The system includes two stages in the workflow: training and prediction. Spark-CRF creates RDDs in nodes and the user-defined Transformation and Action are used for preprocessing, feature extraction, model training and prediction.

### Evaluation metrics

Precision (P), Recall (R) and F-Measure (F) are generally used to evaluate the performance of NER system. They are defined as follows, respectively.

5 $$ \mathrm{P}=\frac{TP}{TP+ FP} $$

6 $$ \mathrm{R}=\frac{TP}{TP+ FN} $$

7 $$ F=\frac{2\times P\times R}{P+R} $$

Here, TP is the number of bacteria named entities that are correctly identified by the model, FP is the number of bacteria named entities which are incorrectly identified by the model, FN is the number of non-bacteria named entities that are incorrectly identified by the model. P represents the precision, R represents the recall rate, and F-Measure is the average of P and R.

## Results and discussion

This article mainly carried out the following two experiments:

In order to verify the classification performance of the proposed Spark version, we choose to compare the proposed system to CRF++ on single node [8] in terms of the P, R and F-Measure on the same datasets. Taking the first 1000, 2000, 3000, ..., and 10,000 sentences of the manual annotated training set [8] to form 10 training sets for model training. The Spark version performs better than the previous results (Table 1). We can also see that with the increasing scale of the training data, the F-Measure increases for both systems on the whole.

**Table 1:** The performance of models trained on different scale training sets

Training set (The number of sentences)	CRF++ on single node			Spark version		
	Precision	Recall	F-Measure	Precision	Recall	F-Measure
1000	84.679%	73.429%	78.654%	86.715%	80.566%	83.527%
2000	85.442%	76.391%	80.664%	88.031%	80.880%	84.304%
3000	86.287%	78.232%	82.062%	88.623%	81.463%	84.892%
4000	85.707%	78.591%	81.995%	88.389%	82.002%	85.076%
5000	86.447%	78.725%	82.405%	88.699%	81.373%	84.878%
6000	87.831%	80.341%	83.919%	89.492%	82.944%	86.094%
7000	88.456%	80.476%	84.277%	89.981%	83.438%	86.586%
8000	87.745%	80.341%	83.880%	90.398%	83.662%	86.900%
9000	88.345%	80.969%	84.496%	90.847%	84.201%	87.398%
10,000	88.873%	81.373%	84.958%	90.944%	83.842%	87.249%

We investigated the effectiveness and scalability of the Spark version by adjusting the scale of application datasets and the number of processor cores. We randomly selected 2000 abstracts, 10,000 abstracts, 20,000 abstracts, 30,000 abstracts, 40,000 abstracts, and 50,000 abstracts respectively in the unannotated texts to form 6 datasets. The number of processor cores is gradually increased from 12 to 48 each time. Each experiment was conducted 5 times repeatedly and the average execution time was recorded.

**Fig. 2:**
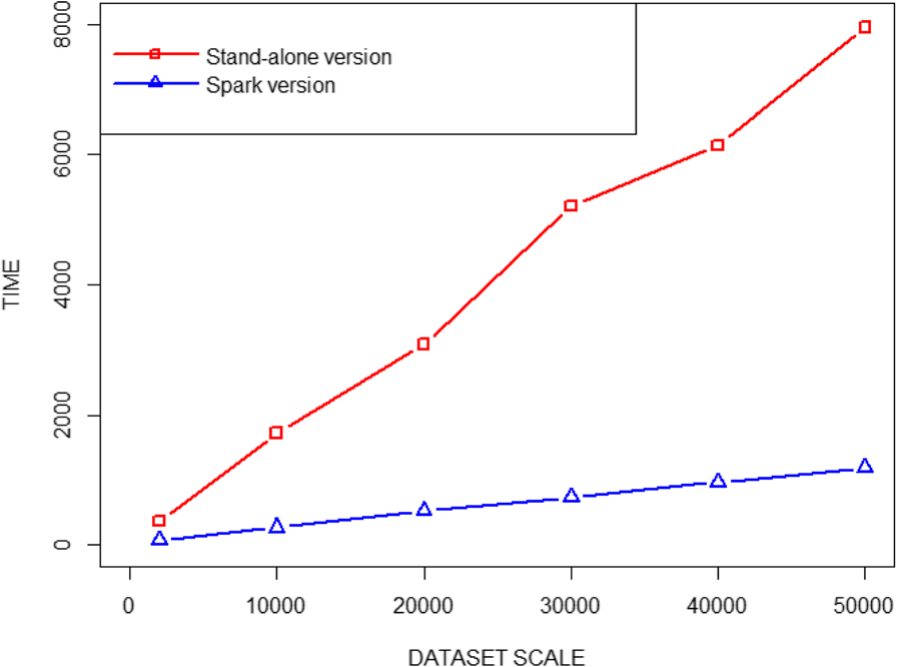
The prediction time and dataset scale curves of CRF++ on single node vs Spark version (48-cores processor)

Table 2 demonstrates that with the increasing scale of the datasets, the average prediction time of both the CRF++ on single node and Spark version is increased accordingly. While the former has many difficulties in dealing with a large amount of data. For different datasets, the prediction time curves of the Stand-alone version and the Spark version (with a 48-cores processor) are shown in Fig. 2. From which we are able to find out that the Spark version runs faster than the CRF++ on single node on the same dataset. With the increasing scale of the datasets, the difference of execution time between the two systems is getting larger and larger and the speed enhancing performance of the Spark version increased significantly. Comparing the prediction time of the stand-alone version and Spark version on the unannotated datasets, it turns out that the speed of the Spark version has been increased by about 3.1 to 6.7 times.

**Table 2:** The average prediction time of CRF++ on single node vs Spark version

Data sets (The number of abstracts)	(s)	Spark version (different numbers of processor cores) (s)			
		12	24	36	48
2000	362.411	118.479	83.758	75.223	72.375
10,000	1716.569	533.486	325.471	286.723	268.614
20,000	3081.027	964.063	612.743	525.29	517.477
30,000	5207.298	1406.216	883.148	793.282	734.974
40,000	6141.149	1858.607	1168.061	1020.059	966.032
50,000	7956.735	2154.872	1465.193	1243.926	1191.362

**Fig. 3:**
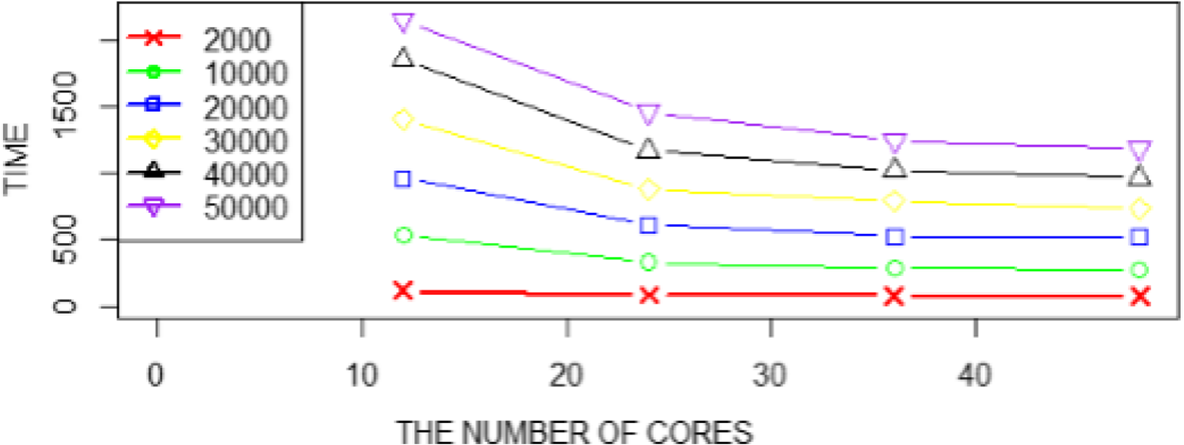
The prediction time and the number of processor cores curves on 6 data sets

The relationship between the prediction time and the number of processor cores on 6 datasets is shown in Fig. 3, which shows that the larger the dataset, the longer the running time under the same number of processor cores; the larger the number of processor cores, the lesser the execution time under the same dataset. This indicates that our proposed Spark version has good scalability.

## Conclusions

This paper provides a computational system of bacteria named entity recognition based on the dictionary and conditional random fields on the Spark platform. The system includes the procedure of text preprocessing, feature extraction, model training and prediction. We also designed experiments to verify the classification accuracy and time efficiency. Under the large-scale dataset, the proposed system is more effective than the previous Stand-alone version (CRF++ on single node). And its efficiency can be further improved with the expansion of cluster computing ability, which shows good scalability. The training sets and test sets used are limited in scale, however, we haven’t verified whether datasets with larger scales would lead to the decrease of accuracy.
